# Telemedicine: Current Impact on the Future

**DOI:** 10.7759/cureus.9891

**Published:** 2020-08-20

**Authors:** Michael X Jin, Sun Young Kim, Lauren J Miller, Gauri Behari, Ricardo Correa

**Affiliations:** 1 Radiology, Stony Brook University Hospital, Stony Brook, USA; 2 Department of Pediatrics, Ann and Robert H. Lurie Children's Hospital, Northwestern University, Chicago, USA; 3 Internal Medicine, Medical College of Wisconsin, Milwaukee, USA; 4 Department of Medicine, University of Arizona College of Medicine, Phoenix, USA

**Keywords:** tele health, tech, public health and social work, internal medicine (general medicine), family medecine

## Abstract

The current healthcare landscape lends itself to major changes, including elevating the prominence of telemedicine. Recent technological advances and external pressures have driven telemedicine to the forefront of medical reality. During an emergency declaration made March 17, 2020, the Centers for Medicare & Medicaid Services (CMS) stated the need for providers to use telemedicine to provide patients care in hospitals, clinics, nursing homes, and other settings across the states. Additionally, new policies have been implemented to better facilitate patient care, safety, and privacy.

The convenience provided by this low resource modality facilitates the intercommunication between physicians and offers a suitable alternative for patients who are medically or socially unable to see providers in person. However, given the nature of the practice, much consideration is needed to build patient relationships and comfort. In the future, the impact of telemedicine on healthcare environments cannot be overstated, especially in hospice and nursing home settings where it stands to improve treatment efficacy and monitoring for the elderly. Newer inventions such as the remote patient monitoring system can act as safety nets for clinic patients, while improving accessibility of electronic health records (EHRs) will dramatically augment available treatment options. However, the spread of telehealth relies on community reimbursement and the ability for physicians to consistently offer the same services that are available in person. Additionally, it is imperative that physicians and other healthcare professionals integrate these new technologies into their fields while also maintaining the ethics of patient security and autonomy.

## Editorial

Background

Telemedicine is the practice of caring for patients over distance. Its use can be traced as far back as 500 BCE, using human messengers to transfer medical advice and medicine. Smoke signals and light reflection were used to communicate medical information such as signaling outbreaks of plagues and notifying births or deaths. Over the past few centuries, the sharing of medical information and telemedicine has advanced through innovations such as the printing press, telegraph, telephone, and Internet. Health care providers are now able to use technology to deliver healthcare directly to a patient in the comfort of their homes using “live-chats” and video calling. Web-based telemedicine services are being tested by healthcare professionals as prospective alternatives for medical regimes. Bluetooth pillboxes can be purchased online to improve medication adherence and convenience. A patient can now obtain limitless medical information with just a few keystrokes, making it both fast and cost-effective. These techniques in the healthcare field will undoubtedly encourage international medical communities to explore the future of telemedicine and address developing global health crises [1].

Current Impact

Recent external factors such as the coronavirus disease 2019 (COVID-19) crisis has minimized direct contact between the people. Subsequently, telemedicine was needed to fill this gap. During an emergency declaration made March 17, 2020, the Centers for Medicare & Medicaid Services (CMS) stated the need for providers to use telemedicine to provide patients care in hospitals, clinics, nursing homes, and other settings across the states [2].

Health Information Considerations

Telemedicine is a low resource modality. To be telemedicine-capable, a provider needs a computer with microphone and camera, smartphone, or tablet with a secure Health Insurance Portability and Accountability Act (HIPAA)-compliant platform to conduct the telemedicine visit [2]. To be HIPAA compliant, video conferencing tools must enable encryption, access controls, and audit logs. Encryption prevents unauthorized access to protected health information (PHI) by masking sensitive data into a format that is unreadable without a decryption key. Access controls utilize unique login credentials to allow actions to be attributed to specific users (who are designated different levels of PHI based on their job roles). Audit logs track who accesses what information and how long they access it for, which allows for unauthorized access to PHI to be detected quickly.

A number of electronic health record (EHR) systems, including Allscripts, Cerner, and Epic, provide basic telemedicine functions. Specifically, HIPAA directs that the vendor must monitor stored data and data during transfer. To meet HIPAA compliance requirements, vendors should provide customers with a business associate agreement (BAA), which takes responsibility for any breaches under their monitoring. In March 2020, the U.S. Department of Health and Human Services (HHS) gave notice that “healthcare providers may use popular applications that allow for video chats, including Apple FaceTime, Facebook Messenger video chat, Google Hangouts video, Zoom, or Skype, to provide telehealth.” HHS also recommended providers notify patients that these third-party applications potentially introduce privacy risks and providers should enable all encryption and privacy modes when using such applications. Although premium services may offer BAAs, basic services for Skype, FaceTime, and Google Hangouts do not offer BAAs. In fact, some services are unwilling to sign a BAA due to the liability. These platforms can be still used in emergency situations but providers will need to be mindful their use may not be in compliance with HIPAA Rules in the future [3].

In addition to the video platform used, HIPAA Rules also must be considered when conducting telemedicine visits because others who can hear or see the video visit may not be visible by the health care provider. Therefore, it is essential health care providers ask patients if there are other individuals in the room that may not be seen on video and obtain permission from the patient for these individuals to be in the “virtual room.”

Patient Safety in Telemedicine

Beyond HIPAA, patient safety is crucial and must be implemented into telemedicine protocols. Patient safety consists of patient identification, confirming a phone number in case of disconnection, obtaining the patient’s physical location in case of an emergency, and confirming emergency contact information. EHR templates can be built to prompt providers to ask and document this safety information. Having the patient’s physical location during the visit (as opposed to their address) is imperative to give to dispatch operators when emergency medical services may be needed. Health care providers should keep this in mind for patients who may participate in their telemedicine visit in a parked car-make, model, license plate number, and location of the parked car will be useful for these emergency situations. Readily available safety information increases health care provider security and confidence during these visits.

Limitations of Telemedicine

The most significant and obvious limitations to telemedicine are the lack of vital sign assessment and limited physical exams. Identifying patients who have tools such as a weight scale, blood pressure cuff, pulse oximeter, and thermometer is helpful. These tools are relatively affordable, sometimes covered by insurance companies, and are often already in the home. Although problems with equipment such as calibration and human use error are possible, health care providers should still utilize these to supplement their data collection to support their clinical decision making. Physical exam through video also should not be discounted as providers can still get a significant amount of information from a visual exam without auscultation and palpation. Health care providers should encourage patients to utilize good lighting and have a flashlight available for their visits. 

Emergency situations can and do arise during outpatient visits and can happen during virtual visits. It is important to have emergency protocols (medical and mental health) in place when conducting telemedicine visits. Obtaining patient safety information as described earlier and having emergency medical services contact information readily available is key. The health care provider should stay connected with the patient by video or phone until emergency medical services arrive and take over, similar to emergency situations that occur in outpatient settings. It is also important to be aware patients may decline advice to go to the Emergency Department or to call an ambulance. In these cases, health care providers should have an American Medical Association (AMA)-type verbal consent available. An example of this consent is: “Per virtual assessment, this patient has expressed decision-making capacity. This patient understands he or she is declining medical advice to go to the Emergency Department (ED) to seek further care and this can result in worsening of symptoms and potentially death. Alternatives to going to the ED include teleflu programs. The patient was advised to call 911 if they experience life-threatening symptoms [3].”

A common concern among providers is the potential degradation of patient-provider relationship. In an early study provided by Dartmouth-Hitchcock Medical center, the quality of dermatologist and patient interaction remain unchanged by the involvement of tele-dermatology equipment [3]. In densely populated regions like India, telemedicine is the preferred method of consultation as hospitals are often under-resourced and health care professionals are scarce. Patients are generally unfamiliar with telemedicine, but once the idea has been elaborated, many had a positive outlook on its potential [2,3]. Building rapport through video or phone is different than when face-to-face, therefore training clinicians on various techniques to improve the patient-provider relationship is essential. An especially helpful tip is looking at the camera when talking to the patient rather than looking at the patient’s picture on the screen because it mimics looking at the patient when talking to them. Clinicians should also openly discuss the differences between telemedicine visits and in-person visits and empathize with the patient if the patient finds it uncomfortable or awkward. Notifying the patient that the virtual visit is only temporary if desired and thanking them for trialing it may improve patient rapport.

Ordering diagnostic tests such as laboratory tests or imaging means a patient still must leave the comfort of their home, arrange for transportation, and come to a clinical setting. As with any care outside of telemedicine, being mindful of diagnostic tests ordered by other providers and consolidating tests needed for a patient to be done in one visit helps take this burden off the patient.

Scheduling telemedicine visits can also be a challenge depending on the scheduling system utilized at the health care facility. Various models of scheduling are being utilized to create a hybrid patient-centered scheduling model to incorporate virtual care into usual face-to-face care models. Health care is starting to parallel our consumer-driven economy. Just as a consumer can choose to get their groceries in person, order online but pick up in person, or have delivered to their home, a patient can now choose to conduct their health care visit in person, by video, or by phone. Some clinics may choose to alternate face-to-face with virtual visits to help decrease patient waiting room times and help increase the time needed to clean patient spaces between patients. This is especially useful in single-provider practices. Other clinics alternate blocks of time allocated to face-to-face or virtual care, which has the added benefits of maximizing teleworking half days or full days. This model is helpful in practices with multiple providers and/or multiple specialties. 

Patient satisfaction can be affected when a telemedicine visit is not enough to address a patient’s health concerns (for example, when diagnostics are indicated that require a patient coming into the facility or when a medication is not prescribed without a face-to-face exam). This limitation should be considered when interpreting patient satisfaction data. Patient satisfaction assessment is still a useful tool and should be assessed routinely to revise telemedicine policies and procedures to maximize the patient experience [3].

Finally, reimbursement for telemedicine visits remains an unknown variable in this virtual modernization of health care. CMS did allow for “Virtual Check-Ins,” which are short patient-initiated communications with a healthcare practitioner. Additionally, Medicare Part B separately pays clinicians for “E-visits,” which are non-face-to-face patient-initiated communications through an online portal. Prior to the COVID-19 pandemic, Medicare only paid for telehealth on a limited basis such as when the person receiving the service is in a designated rural area. As part of the 1135 waiver authority and Coronavirus Preparedness and Response Supplemental Appropriations Act, effective March 6th, 2020 and for the duration of the COVID-19 Public Health Emergency, the Centers for Medicare and Medicaid Services (CMS) stated they will make payment for Medicare telehealth services furnished to patients in all areas of the country in all settings. These visits are to be paid at the same rate as regular in-person visits. It is unclear whether this reimbursement policy will continue and/or how it will change.

Future of Telemedicine

One of the few silver linings of the COVID-19 pandemic is the rise of telemedicine. Telemedicine increases access to healthcare for patients who face barriers such as distance (especially those in rural areas), transportation, or caretaker availability. Immunocompromised patients no longer have to risk acquiring infectious diseases. Patients who wait for months to see a specialist in their geographic region can now see a variety of specialists nationwide and get seen sooner. When a patient inadvertently misses their appointment because of one of these barriers or because they simply forgot about their appointment, a provider can still provide care to the patient using telemedicine, so they don’t have to reschedule, which also decreases missed opportunities and increases clinic efficiency. 

Most of the limitations of telemedicine as described earlier have options and alternatives to make it easier for patients, providers, and facilities. Ultimately, the future of telemedicine relies on the future of telemedicine reimbursement. Providers and patients are getting used to this “new normal” of health care delivery using telemedicine technology, but its sustainability relies on reimbursement.

Regulatory and Legal Implications

To ensure that patient data will be safely recorded, stored, and transmitted while providing telemedicine services, lawmakers will need to concurrently modify both HIPAA and the Health Information Technology for Economic and Clinical Health Act (HITECH). HIPAA and HITECH work synergistically to govern how PHI is managed in this country and will set the future industry standards as telemedicine integrates into healthcare delivery. HITECH promotes the development of infrastructure used to maintain electronic PHI and regulates the goals and uses of health information technology in the United States. HITECH specifically mentions telemedicine technologies to reduce travel requirements for patients in remote areas [3,4]. This language does not fully support the development of telemedicine as a normalized method of healthcare delivery that would be available to all patients, regardless of geographic location. To allow the national healthcare system to fully embrace telemedicine, HITECH needs to define the parameters in which telemedicine can operate within as well as guide the technological requirements that are needed to ensure telemedicine systems are accessible and safe.

While HITECH already contains a provision to include telemedicine in the advancement of healthcare delivery, HIPAA contains no such language. The privacy and security requirements currently defined by HIPAA for the collection, storage, and transmission of electronic PHI can be applied to telemedicine through the current HIPAA-compliance platforms that offer basic telemedicine functionality [3,4]. However, the unique challenges telemedicine will face fall outside of the scope of the current regulatory requirements. To adequately address the new challenges and threats that telemedicine poses to the safety and security of patient data, HIPAA should be amended with a subpart devoted to telemedicine. The amendment should set the minimum privacy and security standards for the health apps and communication platforms used in telemedicine and prevent the storage of patient information on third-party servers. Overall, current HIPAA standards have limited applicability to the future of telemedicine and policy changes need to ensure the federal government has the scope to protect patients [4].

Another regulatory challenge that needs to be overcome is the boundaries of licensing jurisdictions. The virtual visits that telemedicine utilizes allow for physicians to treat patients across state lines. While a physician is typically licensed to practice in their state of residence, that same physician can often be barred from virtually seeing a patient in another state because the state law regards the location of practice as the state in which the patient resides. Telemedicine guidelines vary widely across state lines, and have the potential to prevent widespread implementation of telemedicine services. The Federation of State Medical Boards has already acted to alleviate this burden through the Interstate Medical Licensure Compact (IMLC), which provides an expedited licensure process for physicians who wish to practice across state lines. However, only 19 states currently have implemented the IMLC and other states have failed to adopt legislation to allow for the IMLC to function in their state [4]. Further policy changes will be needed to create a streamlined process for physicians to be licensed for telemedicine across state lines.

Ethics of Virtual Care

As with any major advancement in healthcare delivery, progress cannot be made without reflection upon medical ethics. Telemedicine, as a unique mode of care, requires special attention to ensure ethical standards of practice are not lost. The American Medical Association (AMA) has defined distinct aspects of telemedicine care that physicians need to be responsible for to ensure patients maintain their autonomy and are provided with necessary resources. These responsibilities fall under two main ethical principles: continuity of care and informed consent.

Telemedicine visits are not appropriate for every complaint a patient may present with. Physicians are responsible for discerning the appropriateness of telemedicine and how to direct patients who need to be seen in person, both in non-emergent and emergent situations [4]. Patients should also be encouraged to share their telemedicine encounters with their primary care physicians and should be informed on how to continue their care either with telemedicine or by referral to another physician. Patients should receive the same high-quality care regardless of the mode of care delivery. Any information that a physician would provide to a patient in person should also be provided virtually.

Patient autonomy also should continue to be respected regardless of the mode of delivery. Patients need to be informed of the functionality and limitations of telemedicine services. Physicians should also be obtaining informed consent from the patient or health care proxy as indicated by specialty and institutional practice guidelines. Patients should be given the opportunity to consent to utilize telemedicine before appointments and should be provided the appropriate assistance to access and utilize the technology needed. Additionally, patients should have the option to refuse telemedicine visits to ensure patients are not forced into unwanted modes of healthcare delivery [4,5].

Technological Advancements

One of the biggest advancements coming down the pipeline in telemedicine will be with remote patient monitoring (RPM). RPM allows a patient to wear a device that transmits information to the patient’s phone or tablet and helps them track their health [5]. Examples include automatic insulin pumps, digital blood pressure cuffs, and digital heart rate monitors. The RPM systems allow patients to send their real-time physiologic data to their physicians to streamline the monitoring of the patient’s condition. One of the biggest drivers of the advancement in RPM is patient willingness to adapt to the new technology. A survey released by VivaLNK demonstrated that approximately two-thirds of patients ages 40 and over would be willing to wear an RPM device if it would allow the patient to make fewer trips to the doctor [4,5]. New RPM technologies are rapidly advancing with different monitoring capabilities continuously being released. This trend will continue concurrently with advancements in telemedicine.

An important piece in the expansion of telemedicine will be the integration of telemedicine into current health system workflows and connectivity of telemedicine platforms into EHRs. To maximize the benefit that can be gained from utilizing telemedicine technology, technology, including RPM devices, need to automatically sync to a patient’s chart to allow the physician to have instant access to the patient’s data. The potential onslaught of data that will result from expanded remote monitoring threatens to overwhelm providers if the providers are forced to sift through the data across multiple platforms [3]. The integration of this extra data into EHRs must compromise patient security. Developers should look to guidance through regulatory agencies to include privacy and security protections into the integrated platforms [4,5].

Another predicted advancement in telehealth is the augmentation of user health apps with artificial intelligence. Algorithms built into the app software can detect trends in patient results and prompt the patient to take proactive action before showing symptoms [5]. The apps can also be programmed with bots that will begin eliciting symptoms and other information from the patient as the patient is seeking an appointment and can transmit the information to the provider before the visit. The continued integration of artificial intelligence into telemedicine technology should increase the ease of usability for the apps, increase access to care, and assist patients in adhering to follow-up treatment plans. 

Conclusion

Telehealth has enabled physicians to care for their patients while mitigating the risk of coronavirus infection. Indeed, the capabilities for doctors in both inpatient and ambulatory settings have expanded due to the latitude provided by telehealth. The use of software to connect with patients, elicit a history, and conduct a limited physical exam have proven invaluable tools to aid our healthcare workers. Implementing this technology has decreased direct exposure to PUIs, and in some cases, has obviated the need for certain consulting physicians to enter the patient’s room all together. Workflows for resident and attending physicians alike have been altered to accommodate the absence of direct physician-to-patient contact--moreover, the advent of telehealth has begun to stir a paradigm shift. Specialties such as psychiatry, which rely less heavily on a physical exam, have continued to provide care to patients and make recommendations with minimal alterations in their history-taking. Primary care physicians are adopting telehealth and caring for patients, without either of them having to travel to a clinic. 

The impact of COVID-19 has thrust the nation’s healthcare delivery systems into a new era of advancement centered around telemedicine. Providers rose to the challenge of how to deliver care in a time of social isolation and have proved telemedicine is a viable alternative to seeing every single patient in clinic. While telemedicine cannot yet be used universally for all health care needs, and cannot fully replace an in-person physical exam, rapid technological advancements have the ability to make telemedicine a sustainable method of healthcare delivery, especially to patients who must overcome barriers to accessing care. Even with the unique challenges that telemedicine will have to overcome, the future is virtual.
